# Sensitive SQUID Bio-Magnetometry for Determination and Differentiation of Biogenic Iron and Iron Oxide Nanoparticles in the Biological Samples

**DOI:** 10.3390/nano10101993

**Published:** 2020-10-09

**Authors:** Martin Škrátek, Andrej Dvurečenskij, Michal Kluknavský, Andrej Barta, Peter Bališ, Andrea Mičurová, Alexander Cigáň, Anita Eckstein-Andicsová, Ján Maňka, Iveta Bernátová

**Affiliations:** 1 Institute of Measurement Science, Slovak Academy of Sciences, 841 04 Bratislava, Slovakia; andrej.dvurecenskij@savba.sk (A.D.); alexander.cigan@savba.sk (A.C.); 2 Institute of Normal and Pathological Physiology, Centre of Experimental Medicine, Slovak Academy of Sciences, 813 71 Bratislava, Slovakia; michal.kluknavsky@savba.sk (M.K.); andrej.barta@savba.sk (A.B.); peter.balis@savba.sk (P.B.); andrea.micurova@savba.sk (A.M.); iveta.bernatova@savba.sk (I.B.); 3 Polymer Institute, Slovak Academy of Sciences, 845 41 Bratislava, Slovakia; anita.andicsova@savba.sk

**Keywords:** SQUID, magnetic properties, iron content, magnetite nanoparticles, superoxide, aorta, heart

## Abstract

This study aimed to develop the method for determination of the ultra-small superparamagnetic iron oxide nanoparticle (USPION)-originated iron (UOI) in the tissues of rats on the basis of the magnetic characteristics (MC) in the liver, left heart ventricle (LHV), kidneys, aorta and blood of Wistar-Kyoto (WKY). Rats were treated intravenously by USPIONs dispersed in saline (transmission electron microscope (TEM) mean size ~30 nm, hydrodynamic size ~51 nm, nominal iron content 1 mg Fe/mL) at the low iron dose of 1 mg/kg. MC in the form of the mass magnetisation (*M*) versus the magnetic field (*H*) curves and temperature dependences of *M* (determined using the SQUID magnetometer), histochemical determination of iron (by Perl’s method) and USPION-induced superoxide production (by lucigenin-enhanced chemiluminescence) were investigated 100 min post-infusion. USPIONs significantly elevated superoxide production in the liver, LHV, kidney and aorta vs. the control group. Histochemical staining confirmed the presence of iron in all solid biological samples, however, this method was not suitable to unequivocally confirm the presence of UOI. We improved the SQUID magnetometric method and sample preparation to allow the determination of UOI by measurements of the MC of the tissues at 300 K in solid and liquid samples. The presence of the UOI was confirmed in all the tissues investigated in USPIONs-treated rats. The greatest levels were found in blood and lower amounts in the aorta, liver, LHV and kidneys. In conclusion, we have improved SQUID-magnetometric method to make it suitable for detection of low amounts of UOI in blood and tissues of rats.

## 1. Introduction

Biogenic iron is present in all biological systems. Detail regulation of iron metabolism was described previously [1,2,3]. Nanomaterials, including iron nanoparticles (NPs), are widely used in various industrial applications. However, the fast development of nanotechnologies and nanomaterials may pose a serious health hazard for humans and animals [4]. Ultrasmall superparamagnetic iron oxide nanoparticles of γ-Fe_2_O_3_ (maghemite) and Fe_3_O_4_ (magnetite) with the size of 10–50 nm (ultra-small superparamagnetic iron oxide nanoparticles (USPIONs)) can be used in various biomedical and medical applications [5,6,7]. The advantage of the USPIONs lies in the possibility to use them for targeted drug delivery in the presence of the magnetic field [8]. Stability of USPIONs depends on the local microcellular environment (chemical composition, pH, etc.). Intraendosomal degradation of nanoparticles poses a risk of iron overload, which may be dangerous mainly locally as they can modulate innate iron metabolism on systemic or cellular levels. There is an increasing number of studies that documented intracellular toxicity of iron NPs showing NP-induced inflammation, apoptosis, mitochondrial disorders and oxidative damage [9,10]. Recently, a correlation between exposure to iron oxide NPs and metabolism is of particular concern in nanotoxicology related fields, as NPs can potentially enter to iron metabolism and, thus, to affect its physiological roles. Iron NPs may also increase reactive oxygen species production and to produce oxidative stress, which can further induce adverse effects on DNA, proteins as well as membrane lipids [9,11] and to induce inflammation, changes in blood pressure (BP) regulatory systems via modulation of vascular function. Yet, there is still limited information on the uptake of the USPIONs to the individual organs and tissues and their possible effects on metabolism and physiological functions.

From the methodological point of view, iron content in the tissues can be determined using colorimetric, spectrophotometric, histochemical methods or by the technique of atomic absorption spectrometry depending on the purpose [12,13]. However, these methods do not allow to distinguish clearly between the biogenic iron and USPION-originating iron. Both biogenic nanoparticles (e.g., ferritin) and USPIONs are superparamagnetic, however, usually with the different blocking temperature [14,15].

SQUID magnetometry is a novel approach to quantify different iron forms in biological samples with high sensitivity that may provide new information for the investigation of iron NPs effects on living organism as well as for the understanding of the pathomechanisms of various diseased states. SQUID magnetometry is one of the methods enabling to determine the blocking temperature (e.g., so-called zero field cooled—ZFC, field cooled—FC and alternating current (AC) measurements) [16,17] and in such a way to identify the presence of applied USPIONs. In previous experimental research, SQUID magnetometry was widely used as a tool for determination of the various form of iron. Measurements were done using iron nanoparticles, namely in cell cultures [18,19,20], after in vivo treatment in mice [21,22] and also in embryos of Xenopus Laevis [23]. Using SQUID magnetometry, Janus et al. [24] showed that blood of patients with atherosclerosis was characterised by a higher concentration of ferrimagnetic particles such as Fe_3_O_4_ and γ-Fe_2_O_3_ (associated with the elevated values of the magnetic saturation (*M*_s_)) and significant changes in the superparamagnetic behaviour characterised with changes in the remnant magnetisation (*M*_r_) and the magnetic coercivity (*H*_c_).

The question of the measurement of biogenous iron content is also very important. SQUID magnetometric determination of biogenic iron was performed in various tissues of mice (duodenum, liver, spleen, kidney, heart or brain) [21,25] and rat blood [26]. In addition, iron content was also determined by SQUID magnetometry in human brain [27]. The authors of the above-mentioned studies used various techniques to characterise and determine the amount of iron. Measurement of the *M*(*H*) dependences is the standard method of iron determination by magnetometry, however, more information can be obtained by measurement of the temperature dependences of magnetisation, e.g., the ZFC and FC magnetisation characteristics. Another way is the determination of the isothermal remnant magnetisation (IRM) or using the AC susceptometry.

SQUID magnetometry, in combination with biomedical research, can provide a better understanding of iron metabolism in various diseased states as well as to distinguish biogenic iron from that originating from USPIONs. However, the investigation can be difficult, when NPs are used in very low doses which are diffusely distributed in the human or animal body.

Thus the aim of our study was to develop the method for determination of the amount of the USPION-originated iron in the tissues of rats and to investigate the magnetic characteristics of the liver, left heart ventricle, kidneys, aorta and blood of WKY rats after i.v. application of the low dose of USPIONs.

## 2. Materials and Methods 

### 2.1. Nanoparticles

Commercially available water dispersion of polyethylene glycol (PEG)-coated USPIONs were purchased from Sigma-Aldrich (Bratislava, Slovakia, cat. No. 747408). USPIONs’ concentration was 1 mg Fe/mL and they were dispersed in water. The size of USPIONs confirmed by the transmission electron microscope was 28–32 nm, the zeta potential was −12 mV, polydispersity index was 0.1, and the hydrodynamic size was about 45 nm (all parameters declared by the manufacturer). Before i.v. administration to rats, USPIONs were autoclaved at 121 °C for 30 min and mixed with sterile saline to reach a final dose of 1 mg of Fe/kg of body weight.

### 2.2. Determination of the Iron Core Size, Polydispersity Index and Hydrodynamic Size

**Figure 1:**
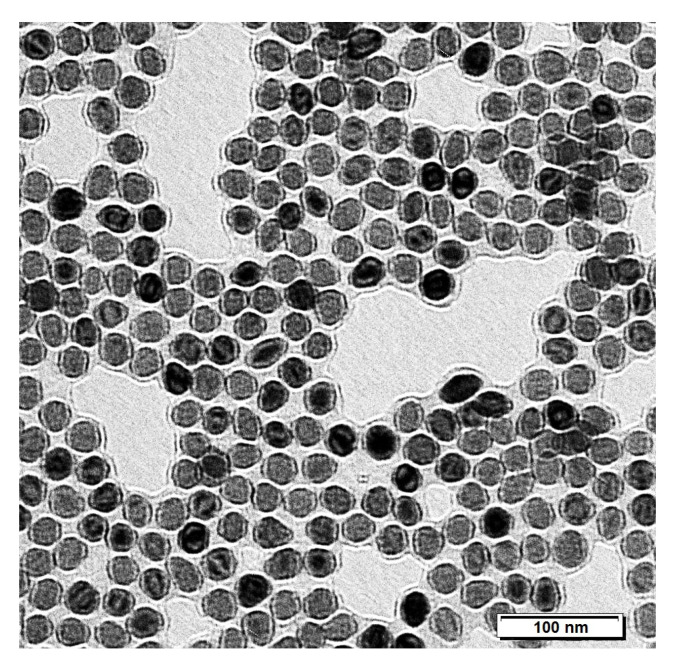
Transmission electron microscope (TEM) figure of polyethylene glycol (PEG)-coated ultra-small superparamagnetic iron oxide nanoparticle (USPION) dispersion. Size of nanoparticles iron core was 29.8 ± 0.2 nm (mean ± standard error of the mean—SEM), and no aggregation of nanoparticles was found.

USPIONs iron core size was checked using transmission electron microscope (TEM) Jeol-1200FX (JEOL Ltd., Tokyo, Japan). As indicated in Figure 1, the average size of USPIONs was 29.8 ± 0.2 nm (mean ± standard error of the mean—SEM), which was in the expected range.

Dynamic light scattering (DLS) measurements were performed using Zetasizer Nano-ZS (Malvern Instruments, Malvern, UK) equipped with a helium/neon laser (λ = 633 nm) and thermoelectric temperature controller at a scattering angle of 173° and 25 °C. All of the data analyses were made in automatic mode. The measured size was presented as the average value of 20 runs, with triplicate measurements within each run.

**Figure 2:**
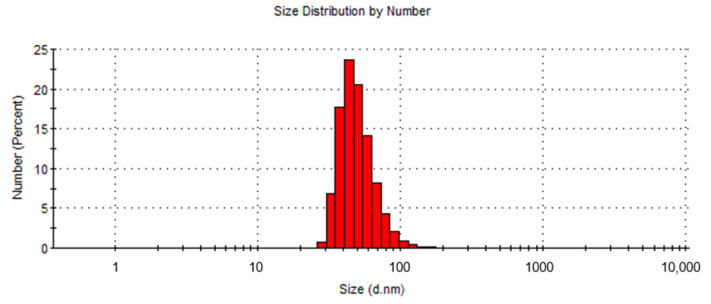
Distribution of USPIONs according to their size.

For the hydrodynamic size analysis, diluted USPIONs (0.01 mg/1 mL) were used. As indicated in Figure 2, the particle size calculated from the number average was 51.3 ± 16.3 d.nm with a dispersity of 0.147. During the measurement, no formation of NPs aggregates was observed.

### 2.3. Animals

All of the procedures used in this study were approved by the State Veterinary and Food Administration of the Slovak Republic in accordance with the European Union Directive 2010/63/EU.

Rats were divided into two groups: control (Cont.) and the group treated with PEG-coated ultra-small superparamagnetic iron oxide (Fe_3_O_4_) nanoparticles (USPIONs). Control rats were given 10-min infusions of saline, starting approximately 30 min from the beginning of the experiment. USPION-treated rats were given 10-min infusions of USPIONs at the dose of 1 mg Fe/kg. 

Wistar-Kyoto (WKY) male rats, 12–16 weeks old, were used in this study. Rats were housed under standard conditions at 22–24 °C in a 12-h light/dark cycle and fed with pelleted diet Altromin formula 1324, variant P (Altromin Spezialfutter, Lage, Germany) and tap water ad libitum.

One day before the experiment, all of the rats had two catheters implanted under 2.5–3.5% isoflurane anaesthesia, as described previously [26]. All of the rats were also pre-treated with meloxicam (Meloxidolor, Le Vet Beheer B.V., Oudewated, Nederland) at 2 mg/kg intramuscularly before surgery to prevent post-surgical pain. Fine-bore polyethylene catheters (Smiths Medical International Ltd., Kent, UK) were inserted into the left carotid artery (internal diameter 0.28 mm) for i.v. administration of USPIONs (suspended in saline) or saline (in control), respectively. Catheters were exteriorised in the interscapular region, and rats were allowed to recover from anaesthesia for approximately 20–24 h. During the experiments, the conscious rats were placed into a plastic box with dark walls and transparent lid (27 cm × 14 cm × 9 cm in size), which allowed the rats free movement. At the end of the experiment, rats were exposed to brief CO_2_ anaesthesia and decapitated 100 min post USPION-infusion. The samples of the liver, left heart ventricle, kidney, aorta and blood were collected for the determination of the magnetic characteristics, histochemical determination of the iron and superoxide production. Tissues were dissected using ceramic scissors and ceramic or plastic forceps. After dissection, the tissues were cleaned out of the connecting tissue, washed in the saline solution and dried of saline solution using filtration paper. Trunk blood was collected into Eppendorf test tubes. Fresh tissues were collected for determination of superoxide and for histochemical analyses. For determination of USPIONs by SQUID, the tissues and blood were frozen in the liquid nitrogen and kept at –80 °C until further analyses.

### 2.4. Superoxide Production

The production of superoxide was measured in the 15–20 mg fresh samples of the tissues using lucigenin (50 µmol/L)-enhanced chemiluminescence using a TriCarb 2910TR liquid scintillation analyser (TriCarb, Perkin Elmer, Waltham, MA, USA), as described previously by Kluknavsky et al. [26]. The results are expressed in the form of cpm/mg of wet tissue.

### 2.5. Histochemical Determination of Iron in the Tissues 

Tissues of the liver, left heart ventricle, kidneys and aorta of control and USPION-treated rats were collected and routinely fixed at 10% buffered neutral formalin. Samples were routinely fixed in paraffin and then cut into 5 µm slices. Slices were deparaffinised and the Perl’s method for iron staining was used for determination of iron in the tissues of USPION-treated and control rats as described previously [28]. Iron was converted to ferric ion with acid solutions of ferrocyanides. Ferric iron in the tissue react with the ferrocyanide, resulting in the formation of a blue pigment called Prussian blue. Nuclei are stained in red by safranine method.

### 2.6. Statistical Analysis

Statistical analysis was performed by Student’s *t*-test. The values were found to significantly differ when *p* < 0.05. The data were presented as mean ± SEM. GraphPad Prism 5.0 (GraphPad Software, Inc., San Diego, CA, USA) was used for the statistical analyses.

### 2.7. Method for Determination of the USPIONs Content in the Tissues 

Determination of USPION content in tissue and blood samples was done by measuring their magnetic properties. A Quantum Design (San Diego, CA, USA) SQUID magnetometer MPMS-XL 7AC was used. Magnetic characterisation of USPIONs was done by measuring the temperature dependence of the mass magnetisation *M* in both the ZFC (zero field cooled) and FC (field cooled) conditions (at the applied magnetic field of 50 Oe), in the temperature range from 1.8 to 300 K and the isothermal magnetisation curves (*M* vs. *H* dependence) measured at the temperatures of 2 and 300 K and the applied field up to 7 T.

#### 2.7.1. The Problems of the Proper Sample Preparing for Magnetic Measurements 

**Figure 3:**
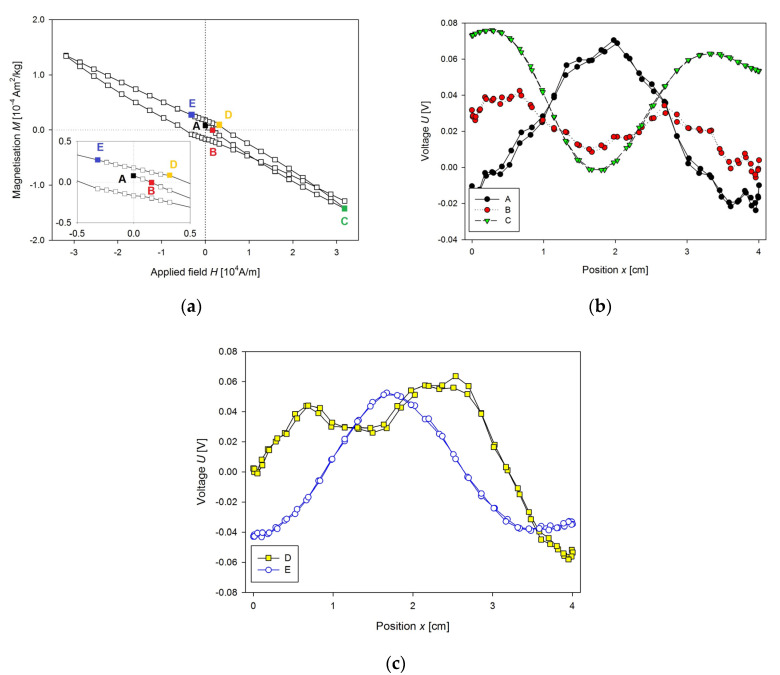
(**a**) *M*(*H*) dependence of the fresh sample of the liver of Wistar-Kyoto (WKY) rat, (**b**) the voltage output of longitudinal SQUID scan for marked points (A–C) of the *M*(*H*) dependence presented in Figure 3a, (**c**) the voltage output of the longitudinal SQUID scan for marked points (D–E) of the *MH* dependence shown in Figure 3a.

At first, we solved the problems of incorrect mounting of the sample. The example of such problematic measurement due to improper sample mounting could be seen in Figure 3a. A sample (103 mg) of the fresh liver of 7-week WKY rat was inserted into standard capsule used in magnetic measurements, and the capsule was fixed by cotton into the straw. *M*(*H*) curve was measured at 300 K. A reciprocating sample option (RSO) was used with a scan length of 4 cm through the 2nd order gradiometer, and the number of averaged scans per measurement was 5. The centring procedure was performed by application of the small magnetic field. The magnet was not quenched, so the start of the measurement was with non-zero magnetisation (Figure 3a, point A). Scan through the gradiometer and voltage output are shown in Figure 3b. Relatively good voltage output was obtained. As the field was increasing (Figure 3a, point B) the magnetisation was changed to the negative values and this way, it was fitted by the MPMS (Figure 3b). Then the maximum field was reached (Figure 3a, point C), this leads to a good fit of the output voltage (Figure 3b) but with unbalanced output on the borders (maximum at 0.5 and 3.5 cm). This unbalanced output results from the position of the sample in capsule and from the cover of capsule and cotton. Then the field decreased to the point D, and its voltage output (Figure 3c) was not properly fitted, giving positive voltage fit of the curve from the sample holder instead of negative one from the sample alone. These false measurements added a hysteresis to the measured *M*(*H*) curve. Point E showed (Figure 3a) no problem with fitting at all, the signal from the sample mounting is again neglected, due to higher output of the sample alone (voltage outputs at the points C and E were rescaled down to be shown with the other signals). There is also a visible problem with sample instability, as the water from the sample was evaporating, the final point of *M*(*H*) curve was not overlapping with the point C.

**Figure 4:**
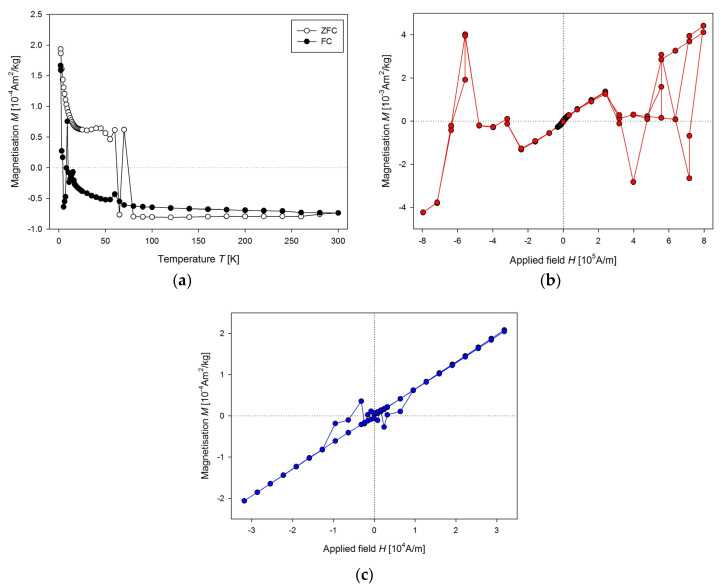
(**a**) Temperature dependence of the mass magnetisation of the same sample as in Figure 3, (**b**) *M*(*H*) dependence of the WKY rat heart at 300 K, (**c**) *M*(*H*) dependence of WKY rat liver at 300 K.

Unsuitable sample holder could also affect the measurement of the temperature dependence of the magnetisation (Figure 4a). When the mass magnetisation goes close to zero, there is significant “jump” to the opposite value, for both ZFC and FC curves. In some cases, the presence of sudden changes in the values of the magnetisation could be observed for higher applied magnetic field too (Figure 4b). Here, the heart from a 9-week old WKY rat was measured. Another example of improper use of a capsule as a sample holder is in Figure 4c. Corrupted measurement occurs in the low magnetic field region, but the overall appearance of the curve is acceptable. 

#### 2.7.2. Polyethylene Holder for Powder and Liquid Samples

**Figure 5:**
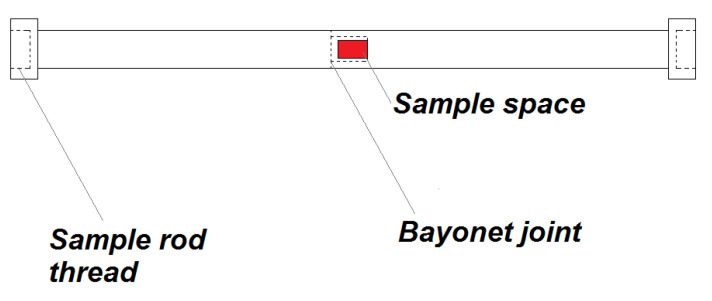
Schematics of high-density polyethylene sample holder configuration.

Considering all the problems and restrictions in the sample preparation for the magnetic measurement, the plastic sample holder (Figure 5) was developed. It is created from high-density polyethylene (HDPE; specification: ultra-high molecular weight polyethylene, Tivar 1000 natural, developed by Quadrant EPP (now Mitsubishi Chemical Advanced Materials Composites, Nitra, Slovakia)). This material shows a relatively small change of magnetic properties with the temperature in the range of 2–300 K, the diameter of the holder is 6 mm and the length of 210 mm was chosen to be as long as a standard plastic straw for MPMS with the end caps. These dimensions were chosen to minimise the weight, possible problems with thermal stability and to minimise the time needed for changes in temperature during cooling and heating. According to [29], the sample space was chosen to be 3 mm in diameter and 5 mm of length.

**Figure 6:**
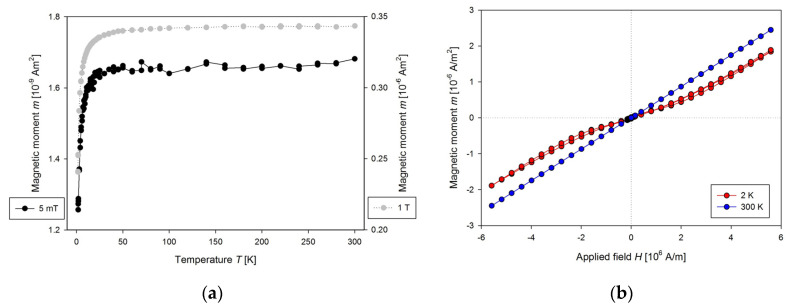
(**a**) The temperature dependence of the magnetic moment for presented plastic sample holder with the cavity filled with He gas measured at 5 mT (left axis) and 1 T (right axis) and (**b**) the magnetic field dependence of the magnetic moment for presented plastic sample holder measured at 2 and 300 K.

If the sample holder was without a cavity for the sample, its length ensures that no signal is created on the output of 2nd order gradiometer. HDPE is diamagnetic, so the cavity for the sample creates proportional signal to its dimensions, but with reciprocal value attributable to paramagnetic material. Magnetic properties of this sample holder with a cavity filled with He gas to minimise the possible paramagnetic output of air are in Figure 6.

The measured data show a strong dependence of the magnetic moment of the cavity on temperature, mainly below 50 K. There is a need to measure corrections for each sample holder before measurement of the samples, which should be later subtracted. The sample holder proved to be temperature stable during measurements, so there is no visible shift in position in the temperature range from 1.8 to 350 K. It is used for measurement of the powder samples and liquids. When measuring such samples, one should be aware of the evaporation of liquid samples during long measurements, as there is often significant change in the magnetic moment of the sample.

#### 2.7.3. Holder and Stabilisation of the Liquid Samples

**Figure 7:**
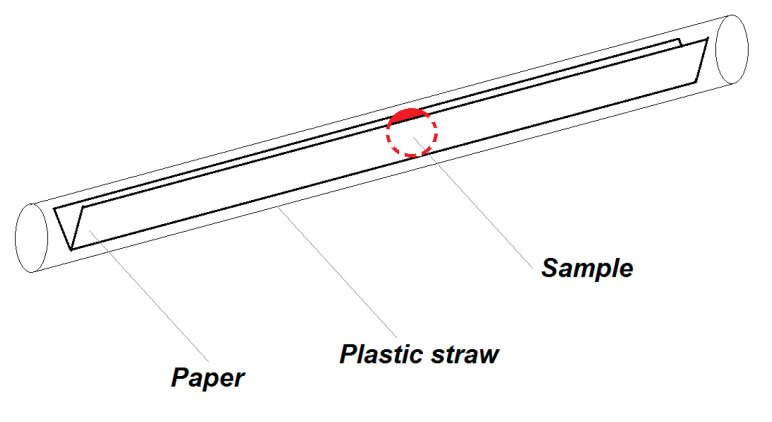
Schematics of sample configuration with V-shaped paper and a standard plastic straw.

For determination of magnetic properties of liquid samples, we use pre-weighted 18 cm long and 6 mm narrow strip of standard office paper (80 g/m^2^), which was bent over the long side to the shape of V (Figure 7), which prevented the sample in the form of drop of 10 µL to spread to sides of the paper. Then the sample was dried in the vacuum for one hour or dried on air for 24 h at room temperature. After drying, the paper with the dry sample was weighted to obtain the actual dry weight of the sample knowing the weight of the paper without the sample. Type of the paper used for measurements of the liquid samples may differ, depending on the experiment, it can be laboratory filtration paper or standard office paper. For nanoparticle dispersion measurement, a strip of transparent foil was used, due to its ability to hold the drop of liquid in the desired shape. After this procedure, the sample was inserted to the plastic straw and attached to the sample rod.

#### 2.7.4. Holder and Stabilisation of the Tissue Samples

**Figure 8:**
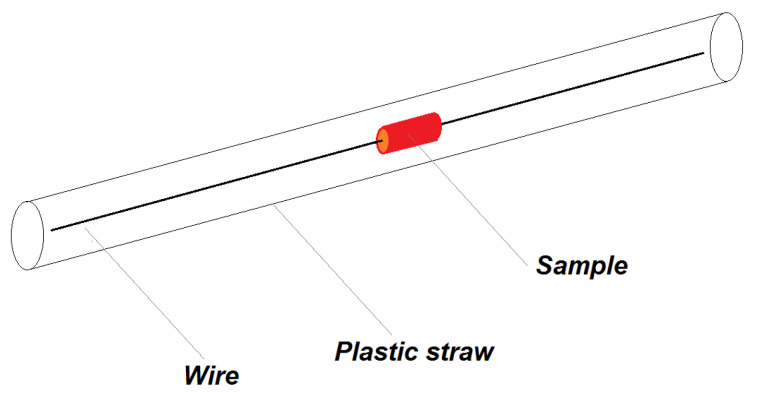
Schematics of the sample configuration in holder with copper wire and a plastic straw.

For measurements of the small tissue samples with the fresh tissue weights about 15–60 mg or even less in case of the aorta (about 10 mg), a method for their stabilisation in the straw holder was derived. Defrosted tissues were cut with cylindrical-shaped instrument with a diameter of 5.5 mm (3.5 mm for kidney) and was mounted on pre-weighted, 18 cm long copper wire with 0.2 mm diameter (Figure 8). The aorta was not cut, it was mounted by a wire embedded via the lumen of the artery. The sample was then vacuum dried for 1 h. During drying the sample shrunk and adhered to the wire. Diameter of the dried sample was then ~4.5 mm (~3 mm for kidney) and the sample attached to the wire was weighted, so we obtained the dry weight of the sample. The Cu wire itself is diamagnetic, long enough again to have negligible output signal to a magnetic moment of the sample.

## 3. Results

### 3.1. Magnetic Characteristics of USPION Dispersion per Se 

**Figure 9:**
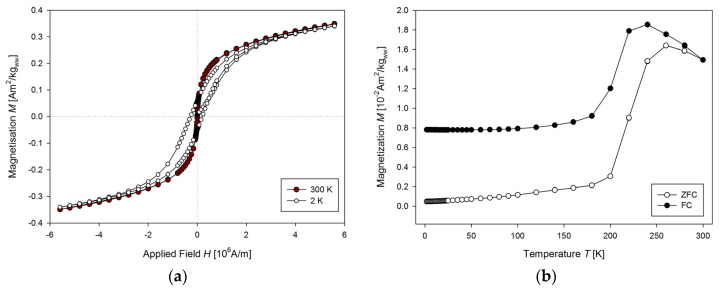
Properties of USPION dispersion per se. (**a**) The mass magnetisation vs. the applied magnetic field measured at 2 and 300 K. (**b**) The mass magnetisation vs. the temperature measured at 4000 A/m applied magnetic field.

To determine USPIONs uptake during the experiment with animals, one need to characterise USPIONs dispersion. A volume of 10 µL of the dispersion was dropped to the transparent foil (18 cm long and 4 mm wide) vacuum dried for 1 h and inserted into the straw as described above. The hysteresis curves were measured (Figure 9a) at 2 and 300 K. The curves show no difference in total magnetisation at 2 K compared to 300 K, and the USPIONs dispersion is superparamagnetic with no hysteresis at 300 K, but the tendency to saturation is present. The temperature dependence (Figure 9b) showed that the ZFC curve has a peak with maximum at 260 K, indicating that at room temperature the NPs are superparamagnetic. The FC curve exhibits maximum at 240 K and a plateau at low temperature, which can be ascribed to interparticle interactions.

### 3.2. Magnetic Characteristics of USPION-Treated Tissues 

**Figure 10:**
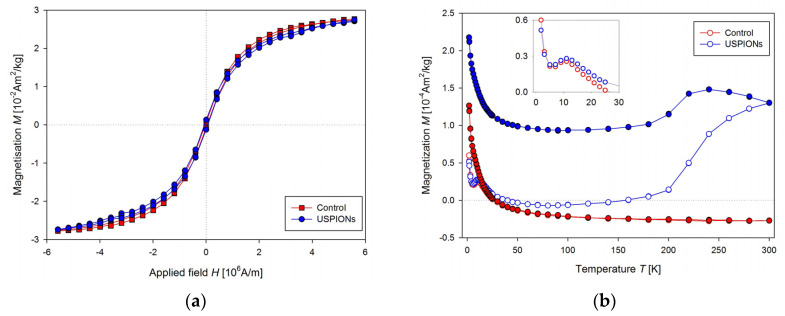
Magnetic properties of the liver of control and USPION-treated rats. (**a**) The mass magnetisation vs. applied magnetic field measurement at a temperature of 2 K, (**b**) the mass magnetisation vs. the temperature at the applied magnetic field of 4000 A/m. The inset shows the peak at the ZFC (zero field cooled) curve both for control and USPIONs group. Curves are the average of 5 measurements per group.

Samples of the liver, left heart ventricle, kidneys, aorta, and whole arterial blood were prepared according to the procedures described above (Section 2). Magnetisation (*M*) measurements were realised similarly to the measurement of USPION dispersion. Hysteresis curves measured at 2 K for control and USPION-treated group, respectively (Figure 10a) showed almost no differences between these two groups, the saturation magnetisation (*M*_s_) and the magnetic coercivity (*H*_c_) were almost the same. However, the measurement of the temperature dependence of *M* (Figure 10b) showed considerable differences between control and USPION-treated group. ZFC and FC curves for the liver of USPION-treated rats were similar to that determined in the USPION dispersion per se at the higher temperature values and confirmed the presence of the USPIONs in the liver. There is also clearly visible maximum for the ZFC curve at 10 K (Inset of Figure 10b), which is characteristic for ferritin and confirms its presence in the liver. Liver of the control group showed only a contribution of naturally occurring ferritin and diamagnetism at the higher temperature (Figure 10b). As the temperature dependence measurement is time and liquid helium (used for cooling the MPMS) consuming, it was decided to measure the contribution of USPIONs at 300 K by measuring shortened *M*(*H*) dependence.

**Figure 11:**
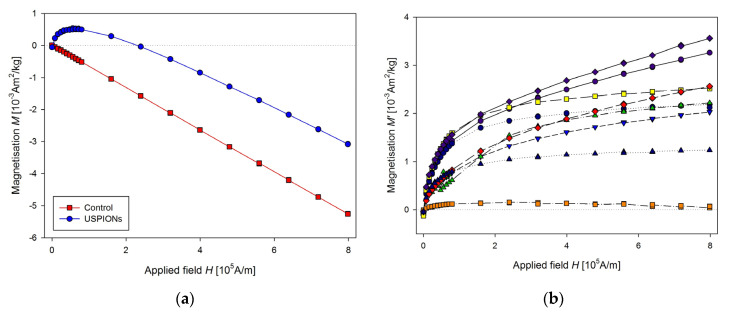
Magnetic properties of the liver of control and USPION-treated rats. (**a**) The partial *M*(*H*) dependences at 300 K. Data are presented as the averaged curve from the control group (*n* = 11) and USPION-treated group (*n* = 9). The mass of dry samples was in the range of 12–30 mg. (**b**) Corresponding *M*’ data for individual rats of the USPION-treated group after subtraction of the control group average.

Parameters of sequence used for measurement are: RSO, scan length 4 cm, 2 × 5 scans per measurement, corresponding to every single point in the figures, frequency 1.5 Hz, measurement length of the partial *M*(*H*) dependences was 20 min, the applied field increased one-way only from 0 to 1 T. This setup, together with improved sample mounting, allows us to perform rapid and precise measurements of magnetic properties of the samples. Result of the measurements of liver samples is shown in Figure 11a. The presented data are averaged of control (*n* = 11) and USPION-treated (*n* = 9) group. In the control group, *M* decreases with the applied magnetic field linearly, which is caused by prevailing diamagnetism of the investigated samples. Data from USPION-treated rats showed initial curvature at low fields, which originated from the USPIONs. After this curvature the slope of the curve goes similar to the control group, indicating the still prevailing diamagnetism in the tissue samples over the contribution of the USPIONs, whose magnetisation saturates at the higher applied magnetic field.

Determination of USPIONs content in the treated tissue was done by subtracting of the averaged value of the mass magnetisation of control group values (*M*_control_) from each of the USPION samples values (Figure 11b):

(1) $${M^{\prime }}_{\mathrm{sample}}=\;M_{\mathrm{sample}}-\;M_{\mathrm{control}}$$

and comparing *M*′_sample_ with the mass magnetisation of the USPION (*M*_USPIONs_) dispersion measured at 300 K and 1 T field. The iron content in the USPIONs treated samples was determined using the following relation:

(2) $$c\left[\frac{µg_{\mathrm{Fe}}}{g}\right]=\;\frac{{M^{\prime }}_{\mathrm{sample}}\;\times \;m_{\mathrm{Fe}}}{M_{\mathrm{USPIONs}\;}\times \;m_{\mathrm{sample}}}\;\times 10^{6}$$

where *c* is the USPIONs content in the sample (in µg of Fe per gram of the sample dry weight), *m*_Fe_ is the mass of iron in USPION dispersion (in our case: *m*_Fe_ = 10 µg) and *m*_sample_ is the mass of the dried sample.

**Figure 12:**
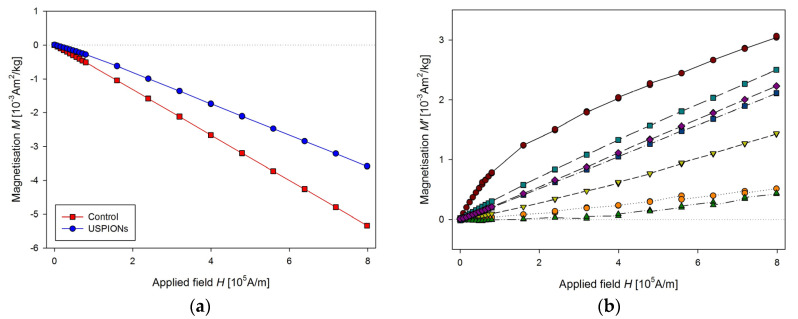
Magnetic properties of the left heart ventricle of control and USPION-treated rats. (**a**) The partial *M*(*H*) dependences at 300 K and (**b**) corresponding *M*’ data for individual rats of the USPION-treated group after subtraction of the control group average. Data (a) are presented as the averaged curve from the control group (*n* = 10) and USPION-treated group (*n* = 7). The mass of dry samples was in the range of 14–25 mg.

**Figure 13:**
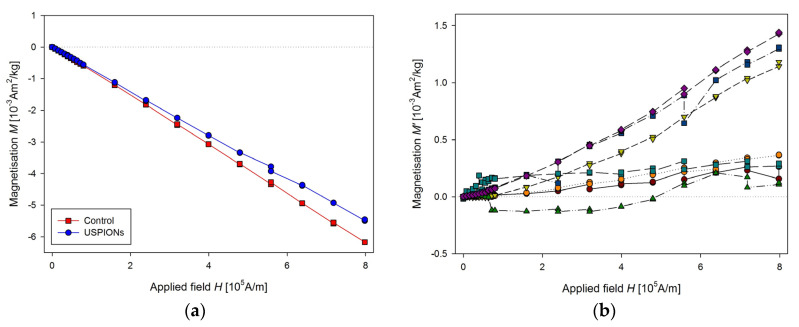
Magnetic properties of the kidney of control and USPION-treated rats. (**a**) The partial *M*(*H*) dependences at 300 K, (**b**) corresponding *M*’ data for individual rats of the USPION-treated group after subtraction of the control group average. Data (**a**) are presented as the averaged curve from the control group (*n* = 6) and USPION-treated group (*n* = 7). The mass of dry samples was in the range of 3–6 mg. The smaller cutting tool was used (diameter of 3.5 mm).

The same procedure of the measurement as for the liver was used for the samples from left heart ventricle (Figure 12) and kidney (Figure 13). In the case of kidney, the smaller cutting tool was used, with a diameter of 3.5 mm.

**Figure 14:**
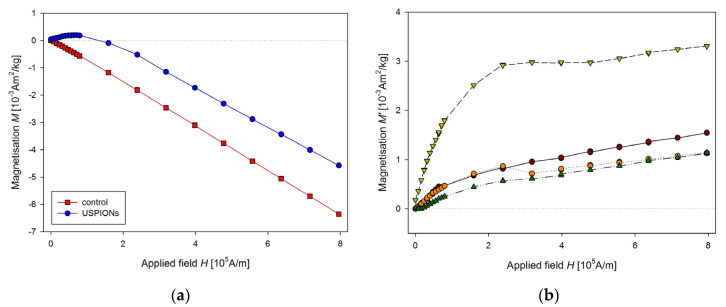
Magnetic properties of the aorta of control and USPION-treated rats. (**a**) The partial *M*(*H*) dependences at 300 K, (**b**) corresponding *M*’ data for individual rats of the USPION treated group after subtraction of the control group average. Data (a) are presented as the averaged curve from the control group (*n* = 4) and USPION-treated group (*n* = 4). The mass of dry samples was in the range of 1.8–2.5 mg.

Samples of the aorta were cut to a length of 5 mm and prepared similarly to tissue samples. The measured mass magnetisation is presented in Figure 14.

**Figure 15:**
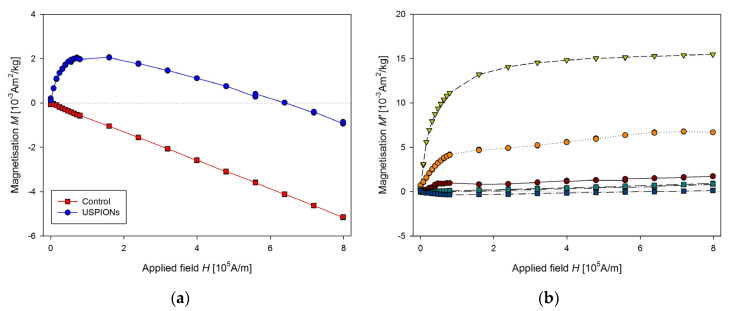
Magnetic properties of the blood of control and USPION-treated rats. (**a**) The partial *M*(*H*) dependences at 300 K and (**b**) corresponding *M*’ data for individual rats of the USPION treated group after subtraction of the control group average (b). Data (a) are presented as the averaged curve from the control group (*n* = 4) and USPION-treated group (*n* = 6). The mass of dry samples was in the range of 1.3–2.3 mg.

Samples of blood were prepared according to Section 2.7, before preparation the blood was defrosted and homogenised using an ultrasonic bath for 60 s (50 kHz, 30 W). Measured mass magnetisation is presented in Figure 15.

**Figure 16:**
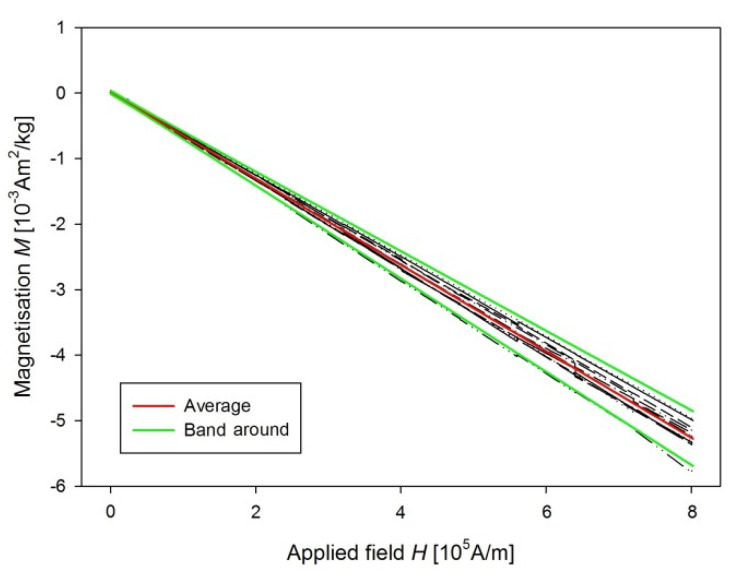
Confidence band around (green lines) of *M* data of the liver control group (black lines for individual samples) and their mean value (red line).

**Figure 17:**
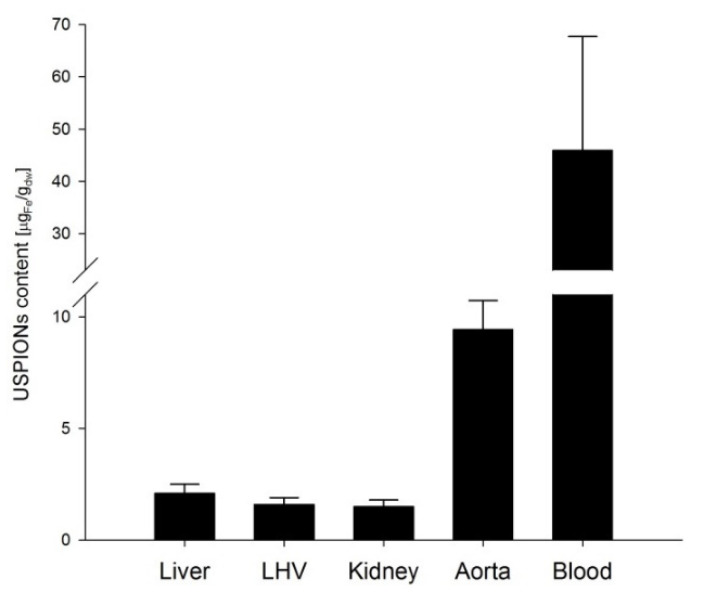
Mean USPION-originated iron content determined in the tissues of liver, left heart ventricle (LHV), kidney, aorta and whole arterial blood. Results are presented as mean ± SEM.

From measurements of *M* of the control groups, we derived the sensitivity of our method of determination of USPION-originated iron content as a variance of measurement changes with the magnetic field (Figure 16). Therefore, a line to the data was fitted by the weighted regression. In particular, we supposed$\;var\left(\epsilon_{i}\right)=\;\sigma^{2}x^{2}$, i.e., the weights $w_{i}=\;1/x_{i}^{2}$. Then, a confidence band around the fitted line $\hat{y}$ is determined as $\hat{y}\pm 2\hat{\sigma }x_{i}$, where the parameters of $\hat{y}$ and $\hat{\sigma }$ were estimated by the weighted least squares procedure. Then the upper border of the band around should be considered as a minimum value of *M* for a sample with USPION content. Value of this minimal iron content was determined using the same procedure as for the USPION treated samples. Determined sensitivity is presented in the Table 1 for each type of samples. The samples with the lower USPION content than the determined sensitivity were omitted and final mean USPION content (for dry and wet sample) is presented in the Table 1 together with number of averaged samples. Presented are also dry and wet weights of the samples of each measured tissue and blood. For better clarity, the data for the USPION content in tissues presented also in the Figure 17.

### 3.3. Histochemical Staining of Iron in the Tissues 

**Figure 18:**
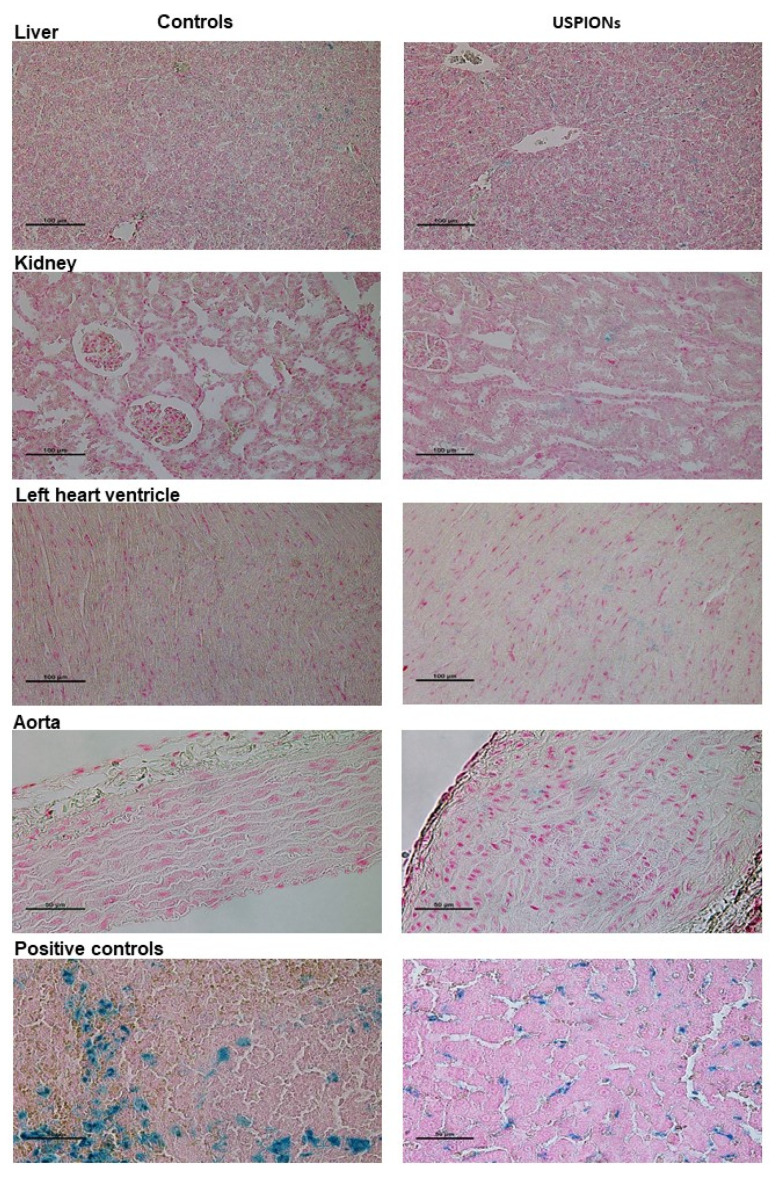
Iron staining in the liver, left heart ventricle, kidneys and aorta of control (left column) and USPION-treated (right column) rats. Staining of iron in the spleen of control rat (bottom left, positive control of biogenic iron) and positive staining of iron in the liver of rats treated with USPIONs at the dose of 20 mg Fe/kg (bottom right, positive control of USPION-originated iron). Prussian blue pigment determines iron. Nuclei are stained in red.

The samples of the liver, left heart ventricle, kidneys and aorta of control and USPION-treated rats stained using the Perl’s method confirmed the presence of iron in the tissues of control and USPION-treated rats. However, it is not possible to unequivocally confirm that iron content in USPION-treated samples is due to USPIONs presence in these tissues (Figure 18).

### 3.4. Superoxide Production 

**Figure 19:**
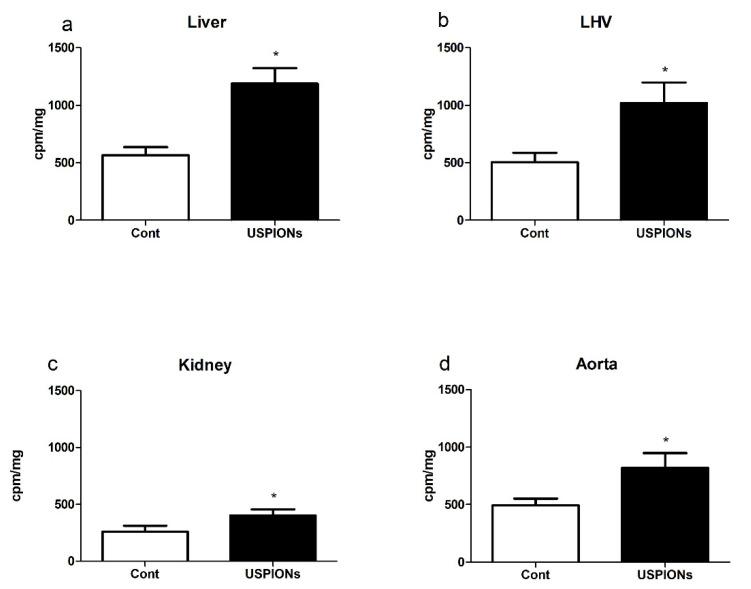
Superoxide production (**a**) in the liver, (**b**) left heart ventricle (LHV), (**c**) kidney and (**d**) aorta. The values represent the mean ± SEM. * *p* < 0.05 vs. control (Cont) group.

Intravenous administration of USPIONs resulted in the significant increase of superoxide production in the liver, LHV, kidneys and aorta by approximately 110%, 101%, 54% and 66%, respectively, compared to the control group (*p* < 0.05 in all groups) as shown in Figure 19.

## 4. Discussion

In this study, we focused on the determination of USPION-originated iron content and its distinction from the naturally present iron in the tissues. For that purpose, we developed the method for determination of the presence of iron originated from USPIONs in the tissues of rats after i.v. administration of a low dose of NPs. We showed that the SQUID magnetometry is able to determine and to distinguish USPION-originated iron even if it is in low amount and not clearly detectable by Perl’s histochemical method. 

We have dealt with the application of USPIONs, and we investigated their effects on various organs of the WKY rats. As bare iron oxide nanoparticles administered in higher doses were previously shown to be toxic as they produce the development of oxidative stress [9,11], we used a low dose of PEG-coated magnetite USPIONs. PEG is a neutral, hydrophilic and biocompatible polymer which improves the dispersion of NPs in water, it improves their bio-distribution and increases blood circulation time [30]. However, despite the PEG-coating and administration of a low dose of USPIONs, we found elevated production of the superoxide, which was increased in all tissues and organs investigated in this study, similarly as it was found using various types of NPs [31]. On the other hand, we were unable to unequivocally confirm elevated iron content in the tissues using the histochemical method. Thus, we used SQUID magnetometry for determination of the distribution and content of USPION-originated iron in the samples of the organs as well as in the blood of rats and for differentiation of USPIONs from the biogenic iron and determination of it content. 

However, due to extremely weak magnetic signals (with changing polarity), it was necessary to analyse and adjust the measurement conditions in the MPMS measuring system. Special attention was paid to preparing and mounting of the samples and to the design of the sample holders. As the magnetic moment of the samples was extremely weak, close to the value of standard holders/capsules, it was necessary to design such a configuration, where the material in vicinity of the measured sample is homogeneous in the whole operating space. In such a way, it is possible to subtract the background from the total measured signal.

We found that the proper mounting of the sample into the MPMS system is extremely important. The frequently used configuration for measurements of biological samples is to use standard plastic or gelatine capsules which are inserted into the drinking-straw and fixed by a cotton stopper [19,21,23,27,32]. However, this configuration leads to several problems. Firstly, there is a need to subtract the background signal generated by the capsule and the cotton. Secondly, when the value of the magnetic moment of the sample is close to that of the capsule, but in the opposite direction, this often leads to instability of scan through gradiometer coils, the usually centred peak is shifted, and the system is not able to properly fit the measured values (as shown in Figure 3). Similar problems were observed in previous studies [33,34], but the authors ascribed them mistakenly as a sort of hardware problem. In some studies, the authors have similar issues with the proper sample mounting. The problem of sample geometry and proper mounting for inorganic samples has been discussed previously in the article of Sawicki et al. [29]. The authors recommended to glue the sample to the long silicone rod attached at the end of the measuring rod for MPMS instead of using a plastic straw. In the study of [25], freeze-drying of the samples and mounting to a straw without capsule was used. In another study [18], the authors described the mounting of cancer cell samples treated with nanoparticles to the long strip of filtration paper. 

Another problem which we have to solve is the attachment of the small samples into the measurement straw. We have solved this problem by the development of the cylindrical shaped instrument to obtain repeatedly the samples of the same shape, because we observed differences in the results if the shapes of the samples were different. Our method with the cutting of the biological samples of the same shape and their attachment during the vacuum drying worked well for samples from the muscles, kidneys or heart, which retain its shape after cutting. The rings of the aorta can also be used when the wire is embedded via the lumen of the artery. However, the samples of the liver or brain were more difficult to be prepared as a cylindrical sample due to their soft structure. In this study, we used a higher mass of the liver to ensure a suitable shape of the sample. With specific caution to the preparation of the sample (cut from the bigger mass of the “crude” sample), our method can also be used for the liver. However, we were unable to perform the measurements successfully in the brain tissues due to their soft structure. We assume that for samples of the brain, their homogenisation in proper buffer and manipulation similar to that for the liquid samples would be more suitable than the procedure for solid tissue samples.

For the liquid samples, such as blood or its derivatives, which need to be prepared differently from solid tissues, we developed a procedure which was inspired with the work of Hashimoto et al. [18]. Liquid samples (10 µL) were pipetted on the suitable type of paper. The type of paper used for the measurement of liquid samples may differ. The laboratory filtration paper can be used for the cell cultures measurement. For blood samples measurement, we used the standard white V-shaped strip of the office (80 g/m^2^) paper, which we found more suitable than laboratory filtration paper. When filtration paper was used, the sample was soaked into it and spread on a relatively big area, which produced inconsistent results. Thus, to keep a small area of the dried sample is essential. Such a preparing of the samples has several advantages: (a) fine localised sample; (b) the sample is time-stable; (c) sample can be stored at room temperature and (d) it is ready for repeated measurements. In addition, as the paper is longer than the base length of gradiometer, the paper has a negligible additional signal to the magnetic moment of the sample. We also assume that this procedure can be used for determination of the presence of the USPIONs in homogenates of the soft tissues that cannot be measured in the solid state.

In the conditions used in our study, we set the detection limit of SQUID magnetometry to 0.6 µg Fe/g of the sample dry weight using PEGylated NPs. Another way of iron NPs determination in rodent tissues has recently been described by Poller at al., who used magnetic particle spectroscopy [35] to analyse fate and metabolic processing of very small iron oxide NPs in atherosclerotic mice after injection of 300 µmol of iron NPs.

It is worthy to note that we observed a relatively high variability of the results during experiment, mainly in blood. This may result from individual metabolic differences between rats. We also found that the amount of USPIONs in blood and tissues can be affected by stress, which significantly alters blood circulation and can also potentially affect the stability of USPIONs [36]. Thus, our experience has shown that caution is needed when manipulating experimental subjects and ensuring the best possible stress-free conditions is needed to reduce the variability of results.

In conclusion, we have improved the SQUID magnetometric method for determination of the low content of nanoparticle-originated iron in the solid and liquid samples of the animal tissues at 300 K, which allows distinguishing the low dose of USPION-originated iron from the biogenic iron naturally present in the tissues and blood. This method is more cost-effective and less time-consuming then measurements at 2 K, which requires liquid He for cooling of the system. Furthermore, the once-prepared sample is stable, and it can be stored at room temperature and repeatedly measured. This method also allows for the determination of the USPIONs in the tissues after administration of the low doses of NPs when standard methods are unable to determine USPION-originated iron in the tissues.

## Figures and Tables

**Table 1:** Summary of USPION-originated iron content in the tissues and whole blood of rats treated with USPIONs (1 mg/kg i.v., 100 min post-infusion). Mean sample mass is presented as dry and wet weight. Sensitivity of the measurement method is expressed in µg of USPION iron per g of dry sample weight. *n* is the number of samples where USPION content was determined. Results are presented as mean ± SEM.

	Sample Wet Weight (ww) [mg]	Sample Dry Weight (dw) [mg]	Fe Content [µg/g_dw_]	Fe Content [µg/g_ww_]	Sensitivity [µg/g_dw_]	*n*
Liver	41.3 ± 2.3	15.9 ± 0.9	2.1 ± 0.4	0.8 ± 0.2	0.6	8
LHV	60.1 ± 4.0	19.2 ± 1.3	1.6 ± 0.3	0.5 ± 0.1	1.1	4
Kidney	13.6 ± 1.7	4.0 ± 0.5	1.5 ± 0.3	0.4 ± 0.1	0.9	3
Aorta	8.5 ± 0.7	2.4 ± 0.2	9.4 ± 1.3	2.6 ± 0.3	1.6	4
Blood	9.6 ± 1.1	1.8 ± 0.2	45.9 ± 21.8	8.4 ± 4.1	1.6	5
